# Recognition and Detection of Concussion in the Community: Implications for Primary Care in the UK

**DOI:** 10.1177/15598276251337429

**Published:** 2025-05-04

**Authors:** Sachin Bhandari, Neil Heron

**Affiliations:** 1 Salford Royal NHS Foundation Trust, Salford, England (SB); 2 Centre for Public Health, 1596 Queen’s University Belfast, Belfast, Northern Ireland (NH); 3 School of Medicine, Keele University, Staffordshire, England (NH)

**Keywords:** concussion, primary care, general practice

## Abstract

Concussion, a subset of traumatic brain injury, is prevalent in both adults and children and can result in a range of short-term and long-term symptoms that can significantly impair an individual’s overall quality of life. Lifestyle factors, such as engaging in high-impact sports may increase the likelihood of sustaining a concussion. Recently, there has been a coordinated effort to raise awareness and educate the general public about the recognition, time-critical interventions, and the associated risks. In the United Kingdom (UK), new grassroots concussion guidance advocates the importance of suspected concussions being diagnosed by a healthcare professional. This will likely lead to an influx of patients presenting to primary care as well as to other medical practitioners, such as those in accident and emergency departments. This review examines and highlights the limitations of the current diagnostic tools available to clinicians in the community and primary care settings. It compares how UK concussion practice relates to concussion practice in the United States (US) and critiques their limitations. It proposes a standardised, computer-integrated tool tailored to the time constraints of UK primary care, pending validation and patient outcome data, to improve diagnostic consistency and efficiency.

‘A standardised tool could offer several benefits, including improved diagnostic accuracy and enhanced consistency in UK clinical practice’.

## Introduction

Concussion, synonymously referred to as mild traumatic brain injury (mTBI), represents a significant public health concern globally.^1,2^ A 2022 report, conducted by the Lancet Neurology Commission, estimated that 50-60 million people worldwide are affected by traumatic brain injury (TBI), resulting in an economic burden of around US$400 billion annually.^2^ In recent years, various initiatives have sought to raise public awareness of concussions and their associated risks. These have included widespread media campaigns, charitable efforts, and the introduction of landmark grassroots sport concussion guidelines in the United Kingdom (UK). Additionally, the latest consensus statement developed in 2022 at the International Conference on Concussion in Sport has further contributed to this effort. These initiatives aim to provide clear guidance, encourage early intervention, and promote a better understanding of concussion. Concussions can occur across all age groups, although the primary mechanism of injury may vary. Amongst the youngest and oldest population cohorts, the majority of concussions can be attributed to falls.^3^ Other frequent causes include sports-related activities, road traffic accidents (RTA), physical assault, and being struck by an object.^1-3^

Much of the current research and guidance on concussion is derived from sports-related incidents, although the challenges identified are applicable more broadly. The latest 6^th^ consensus statement on concussion in sports, while primarily focused on sport-related concussions (SRC), offers a definition that can be relevant to all types of concussions. This definition conceptualises concussion as a ‘traumatic brain injury caused by a direct blow to the head, neck or body resulting in an impulsive force being transmitted to the brain’. Elaborating further, ‘this initiates a neurotransmitter and metabolic cascade, with possible axonal injury, blood flow change and inflammation affecting the brain’.^4^

Failure to recognise and manage concussion in a prompt and timely manner can result in significant cognitive, neurological, and functional deficits that can have prolonged consequences on activities of daily living, thereby impairing quality of life.^5^ In some cases, for example, when an individual suffers a witnessed fall followed by loss of consciousness (LOC) preceded by a clear traumatic head injury, the diagnosis of concussion is relatively straightforward. However, difficulty in diagnosis can arise in unwitnessed incidents, presenting with non-specific symptoms that are often subtle and evolve with time elapsed from initial impact.^3,6^ Currently, there remains no objective gold-standard diagnostic tool, investigation or biomarker that can definitively confirm or rule-out concussion.^3,4^ This poses a significant challenge in concussion management, particularly when presenting to the non-specialist in primary care settings.

## Why is This Relevant to UK Primary Care

An inquiry conducted by the UK department for Digital, Culture, Media and Sport examined ‘the problem of concussion in sport’. This led to the publication of UK grassroots concussion guidance in May 2023. The guidance simplified tailored advice to inform and educate the general public, highlighted the importance of ‘if in doubt, sit them out’, provided timelines for rest and return-to-play protocols, and emphasised the significance of all community-based SRC being seen and diagnosed by a healthcare professional. This will likely lead to an influx of patients presenting to primary care with all types of concussion.^7^ Thus, necessitating the need for a standardised, easy-to-use tool that can be utilised by non-specialist clinicians to accurately diagnose and manage concussion effectively, efficiently, and consistently within the constraints of a primary care consultation, particularly the time-based constraints of typical 10-minute consultations in UK General Practice (GP).

## How Does the United States (US) Compare

Both the UK and US SRC protocols draw from the International Consensus Statement.^7^ However, their approaches differ in scope and implementation. The US Center for Disease Control and Prevention (CDC) HEADS UP initiative targets all levels of sport, from amateur to professional athletes, offers assessment tools such as the Acute Concussion Evaluation (ACE), and is supported by state legislation mandating return-to-play protocols and concussion education in schools.^8^ While there are variations in laws from state to state, it is generally mandatory that individuals obtain clearance to play either from a medical professional, specially selected provider, or any provider trained in concussion management. The Society of Health and Physical Educators America (SHAPE America) provides an overview of how legislation varies between states.^9^ The UK’s grassroots guidance targets community sports, emphasising public education and diagnosis by healthcare professionals without mandating any specific tools or recommendations.^7^ It lacks legislative backing. Both advocate educating the general public to increase concussion awareness, immediate removal of a player with suspected concussion, and graduated return-to-play protocols.^7,8^

Additionally, the CDC has developed a comprehensive guideline on the diagnosis and mTBI among children. The guideline presents 19 recommendations, which are systematically categorised based on the level of supporting evidence.^10^ Routine clinical imaging is generally not recommended for diagnostic purposes of a concussion unless there is a strong suspicion of an intracranial injury, as guided by validated clinical decision rules.^10^ For the diagnosis of paediatric mTBI, healthcare professionals should utilise validated, age-appropriate symptom rating scales within 2 days post-injury and may consider employing computerised cognitive testing within 4 days to enhance sensitivity, while avoiding sole reliance on the Standardised Assessment of Concussion due to its inherent inaccuracy.^10^ Undiagnosed mTBI can lead to delayed treatment and prolonged symptoms, emphasising the paramount importance of effective diagnostic tools in this field.^10^ Further studies are required to assess whether similar multimodal assessment reports such findings within adult populations.

To summarise, the key difference is that the US integrates tools and guidelines into primary care more robustly, backed by government legislation, meanwhile the UK relies on clinical discretion. Whether this results in higher diagnostic sensitivity is yet to be proven; however, it introduces the risk of concussion being under diagnosed in the UK population.

## Short and Long-Term Sequelae

In the short-term, prompt and accurate diagnosis of concussion is imperative. In a sports setting there is a risk of players going back to the field of play with potentially ongoing or evolving cognitive dysfunction that may result in delayed response times and impaired decision-making.^11^ This can increase the risk of further injury, reinforcing the message ‘if in doubt, sit them out’.^7^ Such symptoms are not only limited to the field of play but can in fact translate across all areas of life and increase the risk of additional impact. For example, driving ability may be impaired increasing the risk of an RTA. Additionally, behavioural changes, such as emotional lability, may result in aggressive and hostile behaviour, increasing the risk of engaging in physical violence.^2,3^ The duration of symptoms can vary from person to person. An extended period of symptoms lasting weeks to months can be known as post-concussion syndrome (PCS) and is considered the most common complication.^1^ A major concern in younger children is that sustaining a concussion has the potential to disrupt a critical time for neurodevelopment.^10,12^ Furthermore, if a person is the recipient of a second brain injury in quick succession, without the chance for full recovery, they can suffer a rare but fatal condition where the brain can rapidly swell, and herniation can ensue. This condition, called second impact syndrome, further underlines the necessity for prompt and timely intervention.^1,13^

There remains a paucity of research looking at the longer-term effects of concussion, especially within the paediatric population. However, one of the most notable consequences is the potential development of chronic traumatic encephalopathy (CTE).^1,14^ Most instances of CTE have been reported in association with playing heavy-impact sports, although it has also been linked to other neurotraumatic causes.^15^ From a pathophysiological perspective this condition develops as a result of repetitive head trauma and tau protein deposition in the brain which can trigger a cascade of events leading to progressive neurodegeneration.^15^ Symptomatically this can present with neurological decline, cognitive impairment, memory disturbances, behavioural and mood changes.^1,15^ Quantifying the incidence and prevalence of CTE remains a challenge due to neuropathological examination of brain tissue being the only method of confirming a definitive diagnosis.^15^ Studies of the brains of former American football players found that a resounding 177/202 (87%) of the brains studied were diagnosed with CTE, the severity correlated with the level at which the individual played at. A large proportion suffered with the aforementioned symptoms.^16^ Further research found that the risk and severity of developing CTE correlated with the number of years playing American Football.^17^ Although there is an element of selection bias, these findings suggest a strong association between participation in American Football and CTE development. Recently, in March 2023, a senate inquiry involving National Rugby League (NRL) and Football Australia representatives accepted the link between repeated head trauma and CTE,^18^ indicating a growing recognition of the condition. Furthermore, studies of professional American football and soccer players, acknowledged by the latest consensus statement, found higher mortality rates amongst this population from neurological diseases including amyotrophic lateral sclerosis and dementia.^4^

### Possible Effects on Lifestyle

The short and long-term sequelae of concussion bring the discussion of potential deleterious effects on overall lifestyle to the forefront. Hypothetically, in the short-term, individuals sustaining concussions could be at risk of adverse effects on their physical, mental, cognitive, and social wellbeing. For instance, concussion detection may necessitate removal from play and use of physical aids to help with balance and fatigue. From a mental health perspective, poor emotional regulation, anxiety, and depression may hinder the ability to form and sustain relationships, leading to social isolation and a compounding of the effects. This can result in withdrawal from daily hobbies and activities. From a cognitive standpoint, the potential memory impairments, difficulty in focusing and poor decision-making capability associated with concussion can potentially lead to poor academic performance and challenges in completing workplace responsibilities. These effects may be compounded if an individual suffers from PCS.

A realistic consideration for professional athletes is the potential that a severe brain injury could be career-ending. For example, consider the case of former professional boxing champion, Nick Blackwell, who had to be placed into an induced coma and retire from boxing following a potentially life-threatening brain injury.^19^ Mentally he struggled with being told he couldn’t box again, physically he gained weight and had to re-learn how to walk, talk and eat. He continues to require physiotherapy and reports experiencing fatigue, memory problems, disorientation and confusion. He no longer drives and requires help for activities of daily living.^19^ Such a decision to retire from a sport is underpinned by complexity, necessitates comprehensive evaluation, and input from multidisciplinary teams as well as key stakeholders.^4^ The effects on overall lifestyle have the potential to be drastic.

### Diagnostic Tools in the Community and Primary Care

Currently, concussion is primarily a clinical diagnosis reliant on the expertise of healthcare professionals to discern links between non-specific symptoms in the context of a thorough history and examination. It is important to note that a clinician’s expertise may vary significantly dependent on their previous education, as well as their prior clinical experience in encountering concussions.

Standardised diagnostic screening tools designed to be utilised in the pre-hospital setting by clinicians can help in providing a multimodal evaluation of acute head injuries and determine the presence of concussion. The 6th edition of the Sports Concussion Assessment Tool (SCAT-6) is designed to be used in individuals 13 and older.^4^ There is a child version (Child SCAT-6) designed to be used in children between 8 and 12 years of age.^4^ It is recommended that the assessment is conducted in a quiet setting over at least 10-15 minutes. The tools can be used up to 1 week following the event but are most effective when used within the first 72 hours of sustaining the injury.^4,20^ More recently, an additional Sport Concussion Office Assessment Tool (SCOAT 6/child SCOAT 6) has been developed as a multimodal assessment tool to be utilised 72 hours to 30 days following injury.^21^ However, it has primarily been developed for utilisation by concussion specialists to help in diagnosis and individualising management and takes around 45-60 minutes to complete.^21^ Thus, raising question marks as to whether it would be efficient and effective in routine general practice.

The practical application of these tools reveals potential limitations that require further investigation through peer-reviewed research to determine if they persist more broadly. For instance, the SCAT-6 is heavily reliant on individuals to self-report symptoms (20), which inherently introduces subjectivity. Given the absence of a definitive gold-standard test for concussion diagnosis, this will naturally be present in most tools created. However, it is crucial to recognise and understand the potential issues that can arise, as it may directly impact the accuracy and reliability of findings. For example, in elite sports, individuals may underreport symptoms to expedite their return to play. Additionally, baseline testing is a crucial component for accurate interpretation. In sports with sufficient funding, retrieving past data should not be an issue; however, such data may not be readily available in grassroots, amateur sports, or among the general public, potentially impacting the overall effectiveness for clinicians. Furthermore, components of SCAT-6 can be cognitively and physically taxing to perform. While this may not pose a problem for young, fit athletes, it raises concerns for those with pre-existing physical or cognitive impairments. Moreover, variables including fatigue, stress, and the environment can influence findings, further adding a layer of complexity to interpreting results and potentially affecting the accuracy of the tools. Finally, effective implementation of the tools remains dependent on the clinician’s experience and ability to ensure the tools are used in a consistent and accurate manner. With respect to SCOAT-6, additional challenges emerge. For example, the time-consuming nature makes the tool impractical for use in routine GP appointments. Furthermore, SCOAT-6 requires the clinician to have specialist training or expertise in concussion, which may not be widely available across primary care.

Initially, SCAT-6 was developed to identify SRC. In settings which are less time constrained, where specialist expertise is readily available, and with individuals who have a well-functioning baseline, SCAT-6 can be an effective tool for thorough examination and help to guide diagnosis and management of concussion. However, considering its use outside of a sporting context, possibly due to the lack of alternative concussion screening tools, highlights a significant problem. There is a clear need for a simple and effective tool(s) that can be used broadly across heterogeneous cohorts within a primary care setting.

### A Proposal for Concussion Assessment in Primary Care

A scoping review (pending publication) conducted by the authors identified gaps in primary care concussion tools globally. The review emphasised the need to develop an effective, efficient, and user-friendly validated tool, that could be widely deployed throughout the UK primary care network. Current tools like SCAT-6 are sport-centric and time-intensive. Findings from the review suggest that the tool developed should be integrated into the UK primary care information technology (IT) system and incorporate components from the Brief Concussion Physical Examination (BCPE), the Brain Injury Screening Tool (BIST), symptom-based assessment, and National Institute for Health and Care Excellence (NICE) red flag referral criteria for head injuries (see Appendix 1).^22-24^

An optimal tool would be a 5-7-minute template integrated into electronic medical information system (EMIS) Web, capitalising on GPs’ existing familiarity with the platform. It would provide a standardised approach, requiring minimal additional training or specialist knowledge. The template would estimate concussion likelihood, categorise risk as low, medium, or high, and offer referral guidance, with versions tailored for adults and children. A weighted scoring system, validated in BIST trials,^22^ would minimise subjectivity. A pilot study across a sample of UK practices is needed to confirm sensitivity, alongside a feasibility analysis to evaluate costs and benefits.

A standardised tool could offer several benefits, including improved diagnostic accuracy and enhanced consistency in UK clinical practice. It could facilitate greater data collection and sharing across healthcare institutions, aiding in trend monitoring, concussion recovery management, and future research. Embedding the template in the UK primary care IT system would support precise medico-legal documentation and enable continuous feedback, fostering iterative improvements and supporting community-level initiatives.

## Conclusion

The publication of the latest UK grassroots concussion guidelines, growing media exposure, and increased overall awareness of the risks associated with concussion can be expected to lead to an influx of concussion case presentations to GP, whether that be by self-referral, accessing 24-hour urgent healthcare advice helpline in the UK by dialling 111, accident and emergency (A&E), or through other methods. It is of paramount importance that GPs are well equipped with the fundamental knowledge and tools to effectively diagnose and manage concussions. Currently, despite concussion being considered a relatively uncommon presentation, failure to effectively identify a concussion can have drastic short and long-term consequences, significantly impairing quality of life. Constructively critiquing pitfalls in current care offers the opportunity to iteratively improve the standard of concussion care as a whole, potentially leading to better patient outcomes. It is important that a standardised validated tool which can be widely used by clinicians, regardless of prior experience and expertise, is created to provide a means to diagnose concussions consistently and accurately within the constraints posed by a routine GP consultation. The introduction of such a tool would likely be applicable and benefit medical systems worldwide, including the US. Pilot studies are now imperative to validate this approach, obtain patient outcome data and address its feasibility.

## Supplemental Material

Supplemental Material - Recognition and Detection of Concussion in the Community: Implications for Primary Care in the UK Supplemental Material for Recognition and Detection of Concussion in the Community: Implications for Primary Care in the UK by Sachin Bhandari, and Neil Heron in American Journal of Lifestyle Medicine.
