# Supplementary material for: Recognition and Detection of Concussion in the Community: Implications for Primary Care in the UK
Source: Am J Lifestyle Med. 2025 May 4:15598276251337429. Online ahead of print. doi: 10.1177/15598276251337429 (PMC12052739; doi:10.1177/15598276251337429)
Supplement: Supplemental Material - Recognition and Detection of Concussion in the Community: Implications for Primary Care in the UK [file sj-pdf-1-ajl-10.1177_15598276251337429.pdf]

CONCUSSION SCREENING TOOL

DETAILS

NAME: .... DATE OF BIRTH: .... DATE: ....

DATE & TIME OF INJURY: .... DATE & TIME OF EXAM: ....

SEX: .... ETHNICITY: .... NEXT OF KIN: ....

HAND DOMINANCE: .... CONTACT NUMBER: ....

RED FLAG SYMPTOMS (urgent referral to ED with any of the following)

- ☐ Neck pain or tenderness
- ☐ Seizure or convulsion
- ☐ Double vision or visual disturbance
- ☐ Loss of consciousness
- ☐ Weakness or tingling/burning in >1 arm or legs
- ☐ Deteriorating conscious state
- ☐ Vomiting
- ☐ Severe or increasing headache
- ☐ Increasingly restless or aggressive
- ☐ GCS < 15
- ☐ Visible deformity of the skull

HISTORY

1. Mechanism and events leading to injury

Observe for high-risk indicators such as suspicion of skull fracture , focal neurological deficit, high speed impact, focal blunt trauma or fall from, height (e.g. >5 stairs)

2. Witnesses to the injury and their version of events

Appendix 1:

3. **Have you been sick or vomited**

☐ Yes ☐ No A) If yes how many times ....

If more >1, consider referral to ED

4. **Were you knocked out (or did you lose consciousness)?**

☐ Yes ☐ No A) If yes how many minutes ....

If yes, consider referral to ED

5. **Did you have a fit or seizure straight afterwards?**

☐ Yes ☐ No ☐ Unknown

If yes, consider referral to ED

6. **Are you feeling better, worse, or about the same since in**

☐ Better ☐ Worse ☐ About the same

If worse, consider referral to ED

7. **Have you hit your head or had a concussion/brain injury before?**

☐ Yes ☐ No A) If yes how many times .... B) When was the last injury ....

If yes, act with caution, consider referral to ED

SEVERITY-BASED CHECKLIST (score severity on a scale 0 [Normal] to 6 [Severe]

PHYSICAL	COGNITIVE	EMOTIONAL	SLEEP
☐ **Headache**<br>☐ **Pressure in head**<br>☐ **Neck pain**<br>☐ **Nausea/Vomiting**<br>☐ **Balance problems**<br>☐ **Visual Problems**<br>☐ **Dizziness**<br>☐ **Fatigue**<br>☐ **Photophobia**<br>☐ **Hyperacusis**	☐ **Mental slowness**<br>☐ **Mental fog**<br>☐ **Poor Memory**<br>☐ **Bad concentration**<br>☐ **"Don't feel right"**	☐ **Irritable**<br>☐ **Sadness**<br>☐ **Emotional lability**<br>☐ **Confusion**	☐ **Anxious**<br>☐ **Drowsiness**<br>☐ **Difficultly sleeping**

Fill score in the checkboxes

**TOTAL NUMBER OF SYMPTOMS: /22**	**SYMPTOM SEVERITY SCORE: /132**

PHYSICAL EXAMINATION

GENERAL OBSERVATIONS

Heart Rate: .... Blood Pressure: .... Respiratory Rate: ....

O2 Saturations: .... Temperature: .... GCS: ....

NEWS SCORE: ....

CRANIAL NERVE EXAMINATION

I - OLFACTORY NERVE	▢ NORMAL	▢ ABNORMAL
II - OPTIC NERVE	▢ NORMAL	▢ ABNORMAL
III - OCCULOMOTOR	▢ NORMAL	▢ ABNORMAL
IV - TROCHLEAR	▢ NORMAL	▢ ABNORMAL
V - TRIGEMINAL	▢ NORMAL	▢ ABNORMAL
VI - ABDUCENS	▢ NORMAL	▢ ABNORMAL
VII - FACIAL	▢ NORMAL	▢ ABNORMAL
IX - GLOSSOPHARYNGEAL	▢ NORMAL	▢ ABNORMAL
X - VAGUS	▢ NORMAL	▢ ABNORMAL
XI - ACCESSORY	▢ NORMAL	▢ ABNORMAL
XII - HYPOGLOSSAL	▢ NORMAL	▢ ABNORMAL

If concerned perform a:

- *A full visual examination including fundoscopy, near-point convergence, and accommodation*
- *Tandem gait assessment and examine for focal neurological deficit*
- *Palpation of cervical spine and test cervical range of motion*

Appendix 1: Example guide for a potential tool based on elements combined from previous validated tools including BIST, BCPE, SCAT 6, and the NICE criteria for head injuries [21–24]
